# Synthesis of a New Benzylated Derivative of Rutin and Study of Its Cosmetic Applications

**DOI:** 10.1021/acsomega.4c04908

**Published:** 2025-02-28

**Authors:** Bárbara Janaína Paula da Silva, Ana Cristina da Silva Pinto, Larissa Barbosa Borges, Edinilze Souza Coelho Oliveira, Jullio Kennedy Castro Soares, Lívia Soman de Medeiros, Fernanda Guilhon-Simplicio, Emersom Silva Lima

**Affiliations:** † Faculty of Pharmaceutical Sciences, Federal University of Amazonas, 69077-000 Manaus, AM, Brazil; ‡ Nucleus of Amazonian Micromolecules Studies, Institute of Exact Sciences, Federal University of Amazonas, 690770-000 Manaus, AM, Brazil; § Institute of Environmental, Chemical and Pharmaceutical Sciences, Federal University of São Paulo, 09972-270 Diadema, SP, Brazil

## Abstract

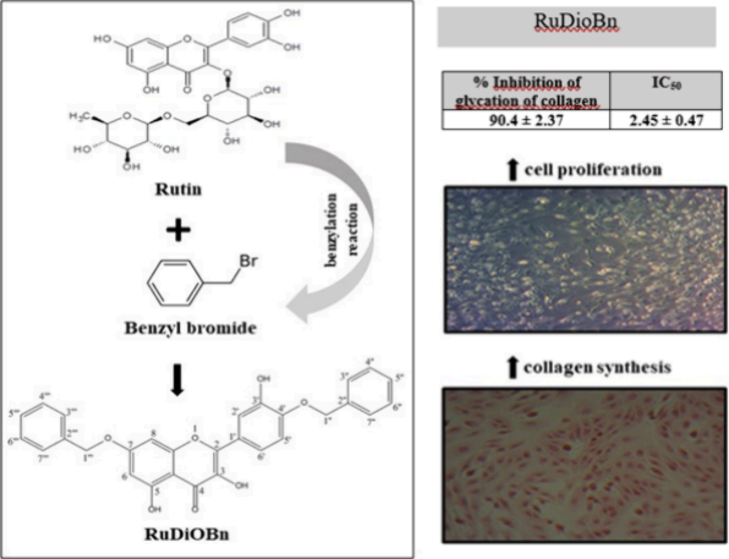

This study aimed to obtain a derivative of rutin that has biological activities for cosmetic applications. The benzylated derivative of rutin was prepared through a substitution reaction of the hydrogens and aromatic rings of rutin by benzylation with benzyl bromide, and the product of this synthesis was encoded as RuDiOBn. The structural elucidation of the compound was performed using NMR and LC-MS/MS. Assays were performed to examine antioxidant (DPPH, ABTS, and cellular antioxidant) content, enzyme and glycation inhibition, cytotoxicity, proliferation, and inhibition of collagen production. In the in vitro glycation assay, RuDiOBn inhibited the formation of advanced glycation end products in collagen via the glyoxal pathway, with an IC_50_ (μg/mL) equal to 2.45 ± 0.47. In the cytotoxicity evaluation, RuDiOBn showed no toxicity to human fibroblasts. Regarding its proliferative activity, there was a significant stimulation in cell proliferation and migration, and it increased the synthesis of collagen deposited in the cell matrix. In the inhibitory activity on collagenase, using the zymographic method, RuDiOBn showed the inhibition of metalloproteinases. Our study presents a benzylated derivative of rutin and aspects of its efficacy and safety for application as a new bioactive cosmetic product.

## 1 Introduction

Skin care has become much more frequent in recent years, principally among young men and women, via the use of cosmetic products for preventing disease or early signs of aging. Aging results from two distinct processes: intrinsic, genetically programmed processes and extrinsic processes, which are caused by external environmental impacts. Despite occurring throughout the body, the most visible signs of aging are those of the skin and most of the change occurs in the dermis, which is mainly composed of a dense extracellular matrix (ECM).^1,2^ In the ECM, aging is associated not only with the thickening of collagen fibrils but also with the disorganization of the total content of this protein due to decreased type I collagen synthesis and increased fibril fragmentation. Type I collagen participates significantly in this process. A decrease in the content of this protein can lead to poor healing.^3,4^

Due to gradual aging and the interest shown by science to control its progress, the search for substances that can control damage to the skin is of great importance. In this context, natural products play a great role in the development of new therapeutic agents and are targets of structural modifications due to their exerting potent effects.^5,6^ Flavonoids, for example, are natural products classified as polyphenols and are found abundantly in the plant kingdom. The flavonoid family is subdivided into classes, where rutin is found (3,3′,4′,5,7-pentahydroxyflavone-3-rhamnoglucoside), which belongs to the subclass of flavanols. In the skin, rutin has an effect on inflammations, such as dermatitis, and helps protect against ultraviolet rays, consequently acting in the reduction of the causes and effects of skin aging and strengthening the elasticity and dermal density through the regulation of enzymes of the ECM.^7,8^

Even with all the evidence of the various benefits in the use of phenolic compounds, in its use, rutin presents low liposolubility and variable bioavailability as its main disadvantage, thus limiting its penetration into a membrane and reducing its pharmacological potential.^9^ To increase the efficacy of this class of polyphenols, several studies have been developed using different strategies of formulations to assist in this problem, such as chemical and enzymatic acylation, nanoemulsions, enzymatic oligomerization, glycosylation, microencapsulation, and microparticles.^10,11^

Researchers have investigated strategies that increase the pharmacological potential of rutin by modifying its chemical structure for the production of new derivatives. For example, Li et al.^9^ performed the synthesis of three new rutin derivatives through enzymatic acylation of rutin with benzoic acid ester, which resulted in improved lipophilicity and antioxidant and anticancer activities. Abualhasan et al.^12^ produced six rutin derivatives based on the ester prodrug strategy, in which the derivatives were formulated with bases for topical ointments and led to a significant increase in skin permeability that facilitated the transport of the active ingredient through biological barriers, thus enhancing its pharmacological effect.

In our study, rutin was structurally modified to create a semisynthetic derivative, through a benzylation reaction, with chemical characteristics aimed at a molecule with pharmacotherapeutic properties superior to the molecule of origin, in order to improve its liposolubility, bioavailability, and effects on skin aging.

## 2 Materials and Methods

### 2.1 Reagents

The rutin used in the synthesis of the derivative was purchased from Sigma-Aldrich, USA, as were the other reagents used in the biological assays. Human fibroblast cell lines (MRC-5) were acquired from the cell bank of the Faculty of Pharmaceutical Sciences (FCF) of the Federal University of Amazonas (UFAM). Dulbecco’s modified Eagle's medium (DMEM) high-glucose culture medium was purchased from Gibco, San Jose, CA, USA, as was fetal bovine serum (FBS) and penicillin–streptomycin.

### 2.2 Rutin Derivative Synthesis

This compound was prepared according to an experimental procedure previously described by Zhang.^13^ Rutin (1.5 g; 2.46 mmol) (Sigma-Aldrich, USA) was weighed into a 50 mL reaction flask, and dimethylformamide (3 mL), potassium carbonate (2 eq-g; 4.92 mmol), and BnBr (2 mL) were added, and the mixture was left stirring and heating (Δ= 60–80 °C) for 24 h. The mixture was concentrated under a vacuum. Ethanol (30 mL) and concentrated HCl (3 mL) were added in succession to the residue and refluxed for 1 h. Product formation was monitored via TLC using hexane/dichloromethane/ethyl acetate (5:1:4). The reaction was terminated by washing the solution with 1% sodium hydroxide, followed by distilled water, and then extracted with ethyl acetate. The organic phase was dried with anhydrous sodium sulfate, filtered and evaporated, and then purified by column chromatography using hexane/ethyl acetate (8:2) as the eluent to provide the product **RuDiOBn.**

### 2.3 Chemical Characterization

The substances were characterized using high-resolution MS and two-dimensional ^1^H, ^13^C, and DEPT NMR. The analyses were performed in the laboratory of the Analytical Center of the Multidisciplinary Support Center (CAM). High-resolution mass spectra were obtained using a quadrupole time-of-flight (QTOF) system, model MicroTOF-QII (Bruker Daltonics, Bremen, Germany), equipped with an electrospray (ESI) source in negative ionization mode. For direct injection MS measurement, the sample solution was introduced at a rate of 180 μL/min with a syringe pump. The QTOF system was calibrated using the sodium formate calibrant with an accepted calibration being <1.5 ppm. Two-dimensional ^1^H and ^13^C NMR spectra were obtained on an NMR spectrometer (Bruker AVANCE III HD) operating at 11.75 t at 500 MHz. Analytical HPLC analyses were performed in a chromatograph (Accela, Thermo Fisher Scientific) equipped with an Accela 600 Pump, Accela Autosampler Plus autosampler, Rheodyne injection valve (25 μL), operating simultaneously with an Accela PDA diode array detector (DAD) and mass spectrometry (MS) (TSQ Quantum Access).

### 2.4 HPLC Analysis

In the present study, after the synthesis and phytochemical isolation of RuDiOBn and the standard rutin, they were subjected to analysis by high-performance liquid chromatography (HPLC). The HPLC studies were carried out at the BioPhar Laboratory of the Federal University of Amazonas. HPLC analysis was performed on high-liquid chromatography instrumentation Instrument Type: Shimadzu LC-10 ATVP, Software: Shimadzu LC Solution, flow rate: 0.800 mL/min, DAD detector: 335 nm, run time: 45 min, injection volume: 10 μL in column dimensions: RP C-18, 250 × 4.6 mm, 5 μm. The mobile phase used was water with 0.2% acetic acid (phase A) and acetonitrile (phase B). The sample was solubilized with Biograde HPLC grade methanol and placed under ultrasound for 20 min for complete solubilization.

### 2.5 DPPH Assay

The assay was performed according to the methodology described by MOLYNEUX (2004),^14^ using the 2,2-diphenyl-1-picrylhydrazyl (DPPH) free-radical scavenging method (Sigma-Aldrich, USA), with some adaptations. In a microplate, 270 μL of DPPH solution (prepared with 2 mg of DPPH distributed in 12 mL of absolute ethanol) and 30 μL of rutin derivative (1 mg/mL) were added. The mixture was incubated for 30 min at room temperature, protected from light. After the incubation period, the absorbance was measured at 517 nm by using a microplate reader (DTX 800, Beckman Coulter, CA, USA). Gallic acid was administered as a standard control, and DMSO was used as a negative control.

### 2.6 ABTS Assay

The assay was conducted based on the methodology described by Re et al.,^15^ with adaptations. The ABTS solution (2,2′-azino-bis-3-ethylbenzothiazoline-6-sulfonic acid, Sigma-Aldrich, USA) was prepared from a reaction involving 0.7 mM radical cleavage in deionized water and 2.4 mM potassium persulfate. The mixture was incubated for 16 h at room temperature and protected from light. For the assay, 30 μL of the derivative was mixed with 270 μL of the ABTS solution, and the microplate was incubated under light for 30 min. After this period, the absorbance was measured at 630 nm in a microplate reader (DTX 800, Beckman Coulter, CA, USA). Gallic acid (Sigma-Aldrich, USA) was used as a standard. Antioxidant activity was calculated using the formula: % antioxidant activity = 100 – (Sample Abs/Medium control Abs) × 100.

### 2.7 Antiglycation Assay

The assay was performed using the bovine serum albumin and glyoxal model (BSA/GO), and a method of measuring anti-AGE activity by oxidative pathway was used, with some modifications, following methodology described by Kiho et al.^16^ A phosphate buffer solution was prepared at 200 mM, pH 7.4, with the preservative sodium azide (3 mM), obtaining a solution of glyoxal at 90 mM and collagen type I at 0.5 mg/mL (Sigma-Aldrich, USA) in phosphate buffer. In the test, 30 μL of the rutin derivative, 135 μL of collagen, and 135 μL of glyoxal were added. Then, the plate was sealed with plastic Parafilm M and incubated in an oven at 37 °C for 4 days. After this, a fluorescence reading was performed (λ_ex_ = 365 nm, λ_em_ = 465 nm) using a microplate reader (DTX 800, Beckman Coulter, CA, USA). Rutin was used as the standard, DMSO as a negative control, and aminoguanidine as a positive control. The experiment was conducted in triplicate. The inhibition percentage was calculated using the equation [inhibition % = 100 – (sample A2 – sample A1/control A2 – control A1) × 100], where A1 is the fluorescence of the initial reading and A2 is the fluorescence of the final reading.

### 2.8 Cell Culture

Human fibroblast cell lines (MRC-5) were obtained from the cell bank of the School of Pharmaceutical Sciences (FCF) of the Federal University of Amazonas (UFAM). At the Cell Culture Laboratory of FCF (FCF/UFAM), cells were maintained in high-glucose Dulbecco’s Modified Eagle Medium (DMEM) (Gibco, San Jose, CA, USA) supplemented with 10% FBS (Gibco, USA) and 1% penicillin–streptomycin (Gibco, USA). Cultivation was performed in an incubator at 37 °C under a humidified atmosphere with 5% CO_2_.

### 2.9 Cytotoxicity

The cytotoxicity assay in fibroblast cell lines (MRC-5) was conducted by the Alamar blue method with resazurin sodium salt (Sigma-Aldrich, USA), following the protocol described by Ahmed et al.^17^ The cells were plated at a concentration of 0.5 × 10^4^ cells per well in 96-well microplates. After 24 h of incubation and cell adhesion, the cells were treated with the derivative. The experiment was conducted in triplicate for each treatment period. As a negative control, the culture medium DMSO was used at 0.01%. As a positive control, doxorubicin was used. After the treatment period, 10 μL of 0.4% resazurin was added (dilution 1:20). The standardized incubation period for the cell line used was 3 h, which is the time required for resazurin to metabolize. After 3 h of incubation, the microplates were analyzed in fluorescence mode (540 nm excitation filter and 585 nm emission filter) using a microplate reader (DTX 800, Beckman Coulter, CA, USA).

### 2.10 Cellular Antioxidant Activity

The evaluation of cellular antioxidant activity was conducted using the methodology of WOLFE and LIU (2007)^18^ based on the detection of intracellular ROS (reactive oxygen species) production through the use of the fluorescent compound 2′7′-dichlorofluorescein diacetate (DCFHDA) (Sigma-Aldrich, USA). In this technique, MRC-5 fibroblastic lineage cells were used, which were sown at a concentration of 6 × 10^4^ cells per well and incubated for 24 h. After this period, the culture medium was removed, and the wells were washed with phosphate-buffered saline (PBS). A DCFHDA solution of 25 μM dissolved in Hanks buffer and 100 μG of the derivative were added to this solution for dilution. An aliquot of 100 μL of this solution was added to the microplate wells, which was incubated for 60 min at 37 °C with 5% of CO_2_. The wells were again washed with PBS and, shortly after, a solution of 2,2′-azobis(2-methylpropionamidine) dihydrochloride (AAPH, Sigma-Aldrich, USA) at 600 μM, dissolved in Hanks buffer, was added to the wells. Then, the microplates were read, and fluorescence was measured at excitation wavelengths of 485 and 520 nm emission for 60 min at intervals of 5 min. As a positive control, rutin was used.

### 2.11 Cell Proliferation

The assay consisted in the evaluation of the growth curve and cell proliferation using Trypan blue (Sigma-Aldrich, USA) according to the methodology proposed by Freshney.^19^ MRC-5 fibroblast cells were plated at a concentration of 3 × 10^4^ cells/mL (DMEM medium, with 10% FBS) in 24-well microplates and kept in an oven at 37 °C and 5% CO_2_. After reaching a cell confluence of 80% of the occupied surface, the cells were treated with the derivative. The proliferation study presented intervals measured at three experimental time points: 24, 48, and 72 h of contact with the derivative. After trypsinization and inactivation with the medium and PBS, 90 μL of the cell suspension was removed and 10 μL of Trypan blue was added. An aliquot of 10 μL of this solution was transferred to the Neubauer chamber, and the cells were counted excluding those stained blue (nonviable cells). As a negative control, the cells were maintained without treatment, containing only the DMEM culture medium with 0.1% DMSO added. As a positive control, ascorbic acid was used.

### 2.12 Cell Migration

In the cell migration experiment, which was carried out according to the method used by Ascione et al.,^20^ MRC-5 fibroblasts were plated at a concentration of 5 × 10^4^ cells/mL in 6-well microplates and maintained in an oven at 37 °C and 5% CO_2_. After reaching a cell confluence of 100% occupied surface, a score was made in the middle of the wells with a 200 μL pipet. The cells were then treated with the derivative. As the standard and negative control, vitamin C and DMSO were used, respectively. Ascorbic acid was used as a positive control. The plate was analyzed using optical microscopy after the treatment intervals of 24, 48, and 72 h. The migration record was made by capturing the image of the microplate with a digital camera (Sony, Alpha NEX DSC E18-55 24.4 2MP 3× optical zoom).

### 2.13 Collagen Synthesis

To quantify the production of soluble collagen in the cell culture supernatant, the picrosirius (Sirius Red) colorimetric assay was used.^21^ MRC-5 fibroblast cells were plated at a density of 1 × 10^5^ cells/mL in 24-well plates and treated with the rutin derivative (100 μg/mL) in triplicate for 24 h. As a control, rutin and ascorbic acid were used. After the treatment time, the cell culture supernatant was transferred to a 96-well plate, which was incubated, without a lid, in an oven at 37 °C overnight for drying the growing medium. An aliquot of 200 μL of the saturated Bouin solution, which had been incubated for 1 h, was added. The fixator was removed, and 300 μL of distilled water was added to each well. The plate was dried at room temperature for 2 h. After this period, 200 μL of 0.1% picrosirius (Sigma-Aldrich, USA) stain was added for 1 h under light shaking and light protection. The dye was removed, and then, 250 μL of HCl 0.01 M was added for the removal of the nonadherent dye. The HCl solution was removed, and then, 150 μL of 0.1 M NaOH was added and maintained for 30 min under mild stirring. The standard collagen curve was prepared from a type I collagen solution from rat tail (Sigma-Aldrich, USA) diluted in PBS and DMEM culture medium without serum. The plate was read on a microplate reader (DTX 800, Beckman Coulter, CA, USA) at a wavelength of 490 nm. Ascorbic acid was used as a positive control.

### 2.14 Inhibition of Collagenase

Collagenase inhibition activity was evaluated using a zymography (gel electrophoresis assay), according to the method described by Salamone et al.,^22^ with some modifications. The MRC-5 fibroblast cells were sown in 12-well plates at a density of 1 × 10^4^ cells/mL. After 24 h, the cells were treated with the rutin derivative. After the period of 24, 48, and 72 h of treatment, the culture medium was collected in an Eppendorf tube in order to perform the SDS–PAGE 1.0 mm gel electrophoresis test using polyacrylamide 30%, containing 10% sodium dodecyl sulfate (SDS), copolymerized with 1.0% gelatin. Each well of the gel was loaded with the derivative at a concentration of 12.5 μg/mL and solubilized in sample buffer (62 mM Tris–HCl pH 6.8; 10% glycerol; 2% SDS; 0.01% bromophenol blue and 25 μg/mL collagenase (obtained from *Clostridium histolyticum*) (Sigma-Aldrich, USA). Electrophoresis was performed with a running buffer (0.025 M TRIS, 0.192 M glycine, and 0.1% SDS, pH 8.5) at a constant 100 V for 150 min. Subsequently, the gels were washed twice for 30 min in 2.5% Triton X-100 (v/v) and then incubated overnight at 37 °C in buffer solution (Tris 50 mM, CaCl_2_ 10 mM, ZnCl_2_ 50 mM). After this period, the gel was stained using the dye Coomassie blue G-250 and bleached with a solution of methanol and acetic acid until the characteristic bands of the activity of collagenases were visible. The evaluation of enzymatic activity was performed by using the software ImageJ.

### 2.15 Statistical Analysis

The results were expressed as mean ± SD (standard deviation from the mean). The means were analyzed using the software <6.0 via two-way ANOVA, followed by the Dunnett test for multiple comparisons with a significance level of *p* < 0.05.

## 3 Results and Discussion

### 3.1 Synthesis and Chemical Characterization

**Figure 1:**
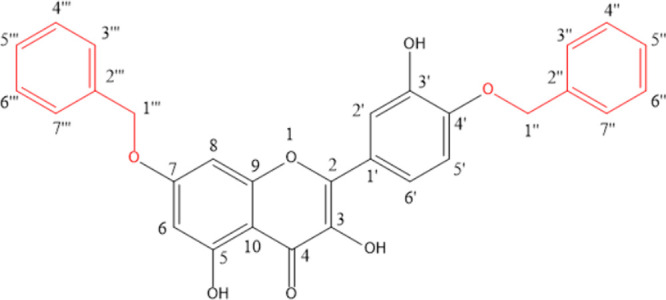
Molecular structure of RuDiOBn.

The product was isolated as a yellow amorphous solid with a yield of 1.1 g (73%), a density of 1.42 ± 0.06 g/cm^3^, an index of refraction of 1.70 ± 0.02, a molar refractivity of 131.9 ± 0.3 cm^3^, and a melting point of 158–161 °C. EI-MS: MF C_29_H_22_O_7_ MW Calcd 482.4808 C (72.19%), H (4.60%), and O (23.21%). The spectrum of total ions, in negative mode, revealed the presence of a base peak of [M-H]^−^ in *m*/*z* 481 and *m*/*z* 390 (loss of Bn-H) (Figure 1S).

**Figure 2:**
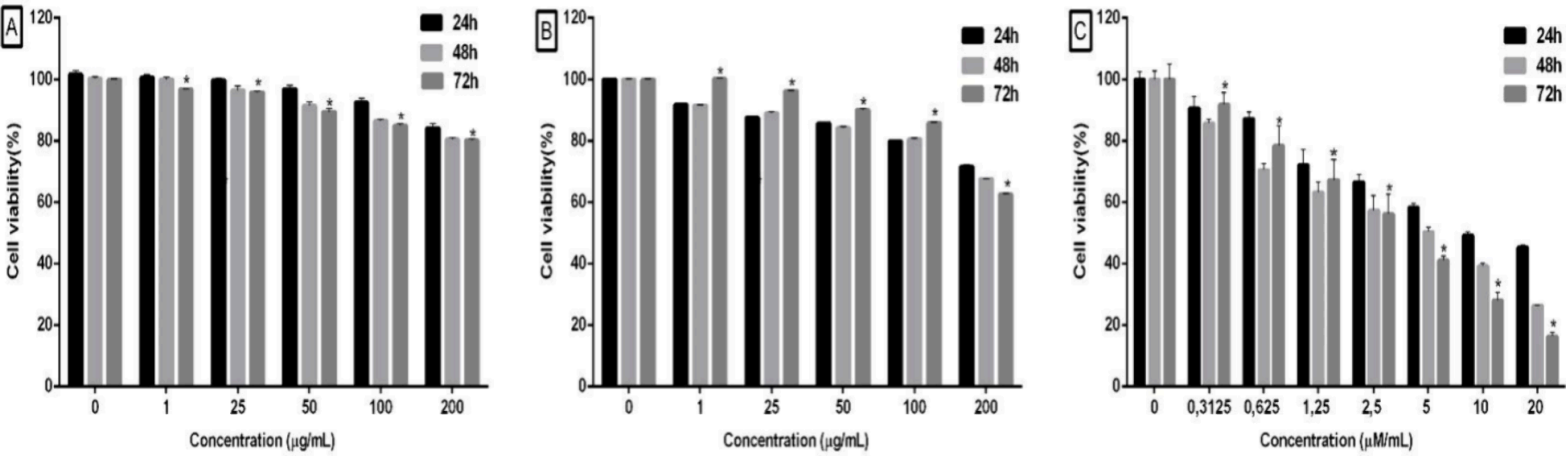
Evaluation of the cytotoxicity of RuDiOBn (A), of rutin (B), and of doxorubicin (C). The tested cells were MRC-5, which were exposed to the test substances for 24, 48, and 72 h. Data are expressed as mean ± standard deviation (relative to DMSO control) and were analyzed using two-way ANOVA, followed by the Dunnett test. *p < 0.05.

In the ^1^H NMR spectrum (Figure 2S), the signals of aromatic hydrogens were observed at δ 6.43 (1H, d, *J* = 2.5 Hz), δ 6.81 (1H, d, *J* = 2.5 Hz), δ 7.76 (1H, d, *J* = 2.5 Hz), δ 7.17 (1H, d, *J* = 8.3 Hz), and δ 7.64 (1H, dd, *J* = 8.3; 2.5 Hz), corresponding, respectively, to the hydrogens H-6, H-8, H-2′, H-5′, and H-6’ of rings A and B of rutin. Signals referring to the aromatic hydrogens of the benzyl groups were observed at δ 7.29 and 7.51 (10H, m), in addition to methylene hydrogen H-1″ and H-1‴ in δ 5.24 (2H, s) and δ 5.21 (2H, s). Signals refer to the hydroxyl groups in δ 12.46 (1H, s, 5-OH), δ 9.44 (1H, s, 3-OH), and δ 7.86 (1H, s, 3′–OH). ^13^C NMR and DEPT data showed shifts of δ 156.9 (C-2), 137.5 (C-3), 177.9 (C-4), 161.4 (C-5), 101.3 (C-6, CH), 164.6 (C-7), 93.8 (C-8, CH), 156.8 (C-9), 105.7 (C-10), 123.0 (C-1’), 115.3 (C-2′, CH), 146.8 (C-3′), 147.9 (C-4’), 113.7 (C-5′, CH), 123.4 (C-6′, CH), 70.3 and 70.8 (C-1″ and C-1‴, CH_2_), 127.8, 128.0, 128.1, 128.3, 128.4, 128.6, 128.9, 129.0, 136.5, 136.6, 137.3, and 137.4 (benzyl aromatic carbons).

**Figure 3:**
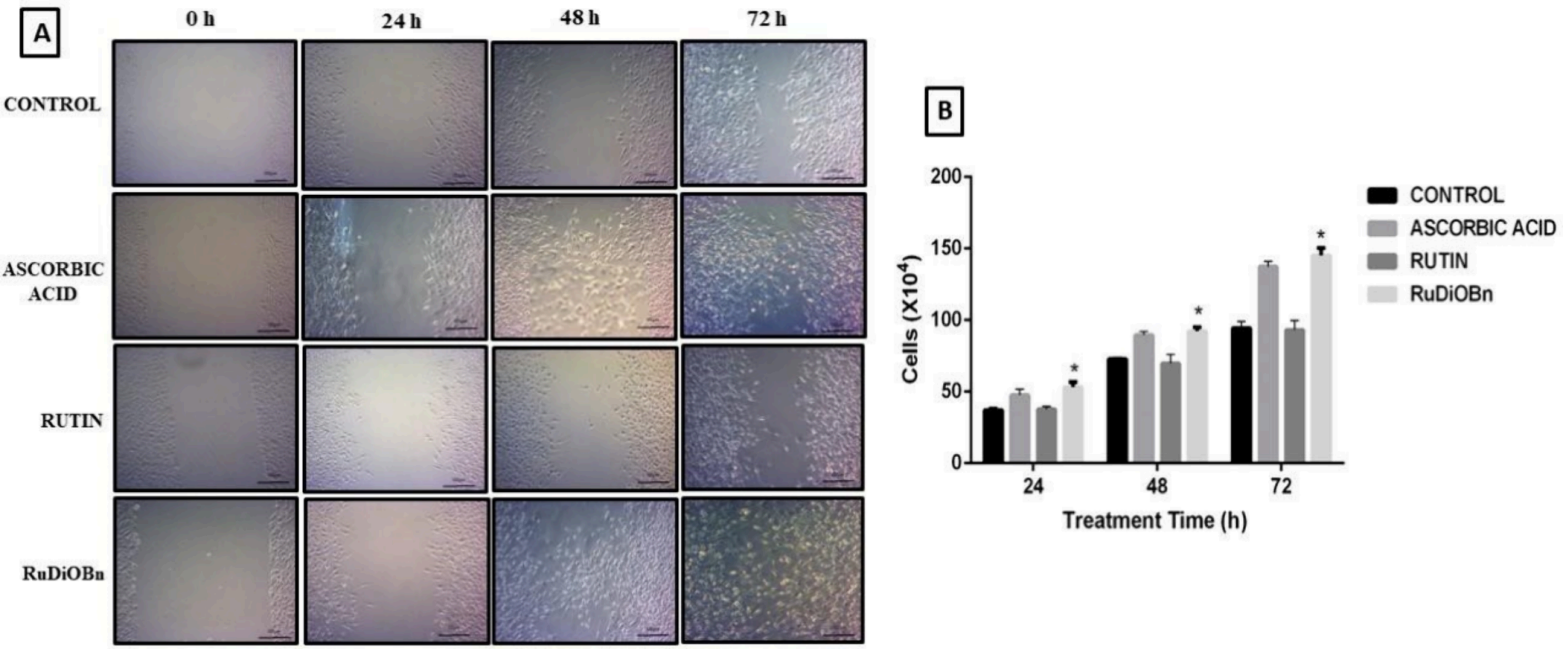
(A) Evaluation of cell migration of fibroblasts exposed to RuDiOBn, rutin, and vitamin C (standard). Photomicrography indicates the migration of cells to the closure of a score, mimicking tissue damage, in a monolayer of cells after treatment with the test substances for the time of 24, 48, and 72 h. (B) Graph of the evaluation of the cell proliferation of RuDiOBn, rutin, and the standard of ascorbic acid in MRC-5 cells, after exposure to the test substances for 24, 48, and 72 h, and these values are represented quantitatively. Data are expressed in mean ± standard deviation and were analyzed using two-way ANOVA, followed by the Dunnett test. * *p* < 0.05 was considered statistically significant when comparing RuDiOBn with rutin.

In the HMBC spectrum (Figure 3S), there was a long-distance correlation of hydrogen at δ 5.21, with carbons δ 123.4, 137.3, and 147.9, and of hydrogen δ 5.24, with carbons δ 127.8, 136.5, and 164.6. Correlation of hydrogen δ 7.86 with carbon δ 146.8. In the HSQC spectrum (Figure 5S), correlations were observed between hydrogen δ 7.76 with carbon δ 115.3, δ 7.17 with carbon δ 113.7, hydrogen δ 5.24 with carbon δ 70.8, and hydrogen δ 5.21 with carbon δ 70.3. Correlations were also observed between hydrogen δ 7.64 with carbon δ 123.4 and hydrogen δ 6.81 with carbon δ 93.8 of the aromatic ring A. The confirmation of hydroxyl substitution in the A and B ring was based on the comparison of data with the literature for benzylated quercetin,^23,24^ with the proposed substance 7-(benzyloxy)-2-(4-(benzyloxy)-3-hydroxyphenyl)-3,5-dihydroxy-4H-chromen-4-one (Figure 1).

HPLC analysis showed the standard rutin with a retention time of 15.20 min and the substance RuDiOBn with a retention time of 29.00 min.

### 3.2 Antioxidant Activity

Based on the analysis of the results presented in Table 1, RuDiOBn showed low antioxidant activity when tested at a concentration of 100 μg/mL, and the percentage of inhibition in all assays was less than 50% when compared with the gallic acid standard (DPPH and ABTS) and rutin. The study of the antioxidant activity of rutin has been evaluated for a long time. Because it is a flavonoid, its potential for inhibiting events mediated by free radicals is driven by its chemical structure due to the presence of multiple hydroxyl groups in the molecular structure.^25^ This structural feature, shared by the aforementioned substance, has been modified and is possibly related to the antioxidant inability of the derivative since RuDiOBn has two hydroxyls substituted at positions 7 and 4’.

**Table 1:** Evaluation of the Antioxidant Activity of the Quercetin Derivative^a^

sample	DPPH	ABTS	cellular antioxidant
RuDiOBn	13.20 ± 3.74	5.90 ± 1.45	2.50 ± 2.77
gallic acid	86.10 ± 1.19	99.80 ± 0.18	–b
rutin	75.10 ± 0.62	85.50 ± 0.81	79.90 ± 1.29

a Study of antioxidant activity via DPPH, ABTS, and cellular antioxidant assays. All substances were tested at a concentration of 100 μg/mL, and the data expressed as a percentage of inhibition with SD of the mean.

b –, not applicable.

### 3.3 Inhibitory Activity of the Glycation of Collagen

Glycation is a nonenzymatic reaction with reducing sugars and amino groups of proteins that undergo structural rearrangements and cause the formation of advanced glycation end products (AGEs).^26^ The accumulation of AGEs is a primary factor for aging because they directly affect long-lived proteins, such as collagen, which are formed and agglomerate throughout life, causing intermolecular cross-links that lead to increased rigidity and reduced skin flexibility.^27^

Table 2 refers to the analysis of the inhibitory activity of the glycation of collagen by RuDiOBn compared to rutin or aminoguanidine (as the positive control). In the formation of AGEs in the glyoxal pathway, RuDiOBn showed significant inhibition at a concentration of 100 μg/mL, exhibiting 90.4 ± 2.37% inhibition of AGE formation, which was higher than rutin and the positive control. In view of the results shown here, the derivative was successful in inhibiting the glyoxal pathway and prevented the binding of glyoxal with the amine group present in collagen, thus contributing to the nonformation of exacerbated AGEs. According to Khan et al.,^28^ rutin is able to prevent the synthesis of AGEs since it encompasses a vicinal dihydroxyl group, which helps in the elimination of ROS and prevents the link between this reactive carbonyl species and the amine group present in collagen, thus making it more efficient when compared with aminoguanidine.

**Table 2:** Evaluation of the Inhibitory Activity of the Glycation of Collagen

sample	inhibition (%)	IC_50_ (μg/mL)
RuDiOBn	90.40 ± 2.37	2.45 ± 0.47
rutin	74.10 ± 2.25	4.63 ± 0.64
aminoguanidine	86.80 ± 2.73	5.70 ± 3.45

Glycation of collagen was inhibited via the formation of AGEs via the glyoxal pathway. Data are expressed as a percentage of the inhibition of the glycation of collagen with mean ± standard deviation (tested at a concentration of 100 μg/mL) and the inhibitory concentration at 50% via a curve with seven concentrations (100, 50, 25, 12.5, 6.25, 3.125, and 1.5625 μg/mL).

### 3.4 Cytotoxicity

For the analysis of possible cytotoxicity, a cell viability assay was performed using the AlamarBlue method. In this, MRC-5 cells were exposed to RuDiOBn during a period of 24, 48, and 72 h with a curve of concentrations of 200, 100, 75, 50, 25, and 1 μg/mL. As a positive control for cell death, doxorubicin was used at concentrations of 20, 10, 5, 2.5, 1.25, 0.625, and 0.3125 μM/mL.

The cells treated with RuDiOBn during 24 h exhibited a percentage of 84.1 ± 0.94 viable cells. At 48 h, the percentage was 80.5 ± 0.95, and at 72 h, the percentage was 80.1 ± 0.91 when tested at a concentration of 200 μg/mL (Figure 2A). Rutin was tested under the same conditions as RuDiOBn and presented a percentage of viable cells of over 60% (Figure 2B). Doxorubicin (standard for cell death) at 20 μM/mL exhibited 45 ± 0.7% viable cells at 24 h, 26.3 ± 0.1% at 48 h, and 16.3 ± 1.7% at 72 h (Figure 2C).

Based on the analysis of the results obtained, it is possible to conclude that RuDiOBn does not present cytotoxicity at the concentrations to which the human fibroblast cell line was exposed.

### 3.5 Cell Proliferation

To evaluate a possible stimulus of RuDiOBn in fibroblast cell proliferation, the Trypan blue assay was used to analyze the amount of viable cells. This method stains dead cells because the cell membrane of viable cells remains intact, preventing the penetration of the dye into the intracellular medium; as a result, viable cells are colorless, while nonviable (dead) cells become blue in color.^29^

After 24 h, the RuDiOBn derivative showed a value of 53 × 10^4^ cells, which is higher than that of rutin (37.5 × 10^4^) and ascorbic acid (47.5 × 10^4^). After 48 h of treatment, RuDiOBn continued to demonstrate an increase in the number of cells, exhibiting 92.1 × 10^4^. However, after 72 h, there was a significant increase in the cells exposed to RuDiOBn, presenting 145 × 10^4^ cells, when compared with rutin (93 × 10^4^) and ascorbic acid (137.2 × 10^4^). As such, RuDiOBn presents a proliferative stimulus to the fibroblast lineage (Figure 3).

### 3.6 Cell Migration

The in vitro migration assay is a complement to the cell proliferation test, which consists of the evaluation of the migratory activity of fibroblasts treated with RuDiOBn, rutin, or vitamin C (standard), after the formation of a stria in the fibroblast monolayer, thus mimicking tissue damage. This analysis was carried out over 24, 48, and 72 h with 100 μg/mL of each substance.

For 24 h, the cells that were treated with ascorbic acid and RuDiOBn began to stimulate migration since their edges appeared thicker. This is confirmed after 48 h with the cells migrating to the center of the score and promoting a more marked closure than the cells treated with rutin. After 72 h, the rutin and the negative control did not show significant migration, while RuDiOBn and ascorbic acid showed closure of more than 90% in the score area (Figure 3). Gegotek et al.^30^ proved that rutin stimulates wound healing through the proliferation of fibroblasts and consequently by the generation of ECM involving collagen synthesis.

### 3.7 Collagen Synthesis

**Figure 4:**
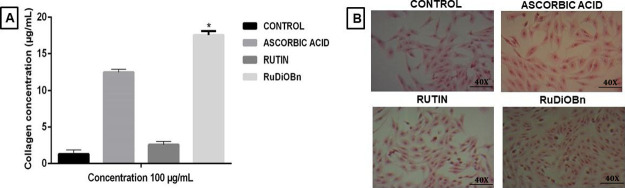
(A) Evaluation of collagen synthesis in fibroblasts stimulated with 100 μg/mL of RuDiOBn, rutin, and ascorbic acid. The data are expressed as collagen concentration in μg/mL, with mean ± standard deviation and were analyzed by two-way ANOVA, followed by the Dunnett test, *p < 0.05, which was considered statistically significant when comparing RuDiOBn with rutin. (B) Photomicrographs of collagen deposited in the ECM of fibroblasts after stimulation with ascorbic acid, rutin, and RuDiOBn.

RuDiOBn was also evaluated to ascertain its potential in collagen synthesis in a fibroblast cell culture. This quantification was performed by using the Sirius Red method. For this analysis, a curve was made with type I collagen to calculate the quantification of collagen synthesized in the fibroblasts. After treatment of the cells with RuDiOBn, rutin, and ascorbic acid (positive control) (100 μg/mL), collagen synthesis was measured in μg/mL, by which 17.5 ± 0.21 was achieved in the treatment with RuDiOBn, which was 2.6 ± 0.05 with rutin, 12.5 ± 0.08 with treatment using ascorbic acid, and 1.3 ± 0.01 mg/mL with the negative control (Figure 4).

The results indicate an increase in the total amount of collagen deposited by fibroblasts through stimulation with RuDiOBn compared to rutin, and this was superior to that with ascorbic acid and the positive control. This data is represented qualitatively in the photomicrographs of Figure 4, in which the absorption of the Sirius Red occurred in the cells after treatment, marking the concentration of collagen deposited by the fibroblasts through stimulation with the test samples. Through quantitative and qualitative analyses, it can be said that RuDiOBn aids the synthesis of collagen.

### 3.8 Inhibition of Collagenases

**Figure 5:**
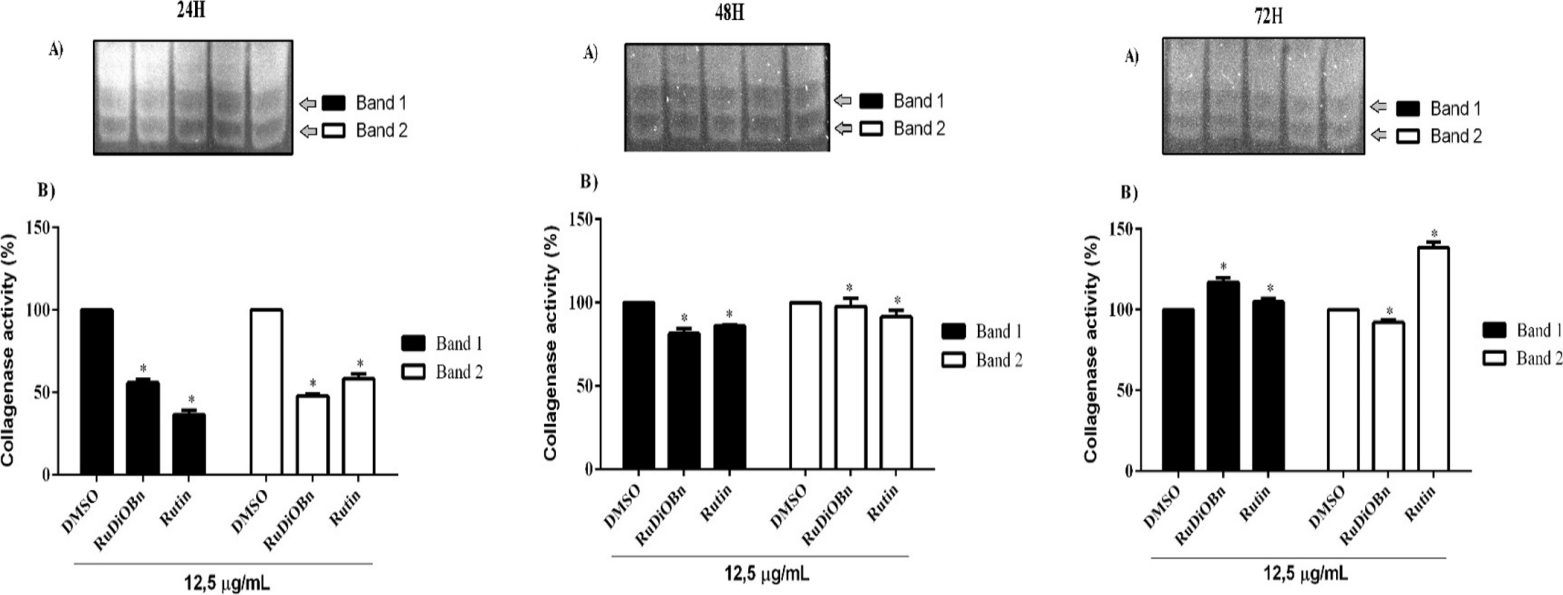
Evaluation of the effects of RuDiOBn and rutin on the inhibition of Clostridium hystoliticum collagenase activity. (A) Photo indicates collagenase bands 1 and 2. (B) Graphs of inhibition of collagenase bands 1 and 2 by the substances (12.5 μg/mL) after collection of the supernatant of MRC-5 cells at the intervals of 24, 48, and 72 h. The analysis was performed using ImageJ software. Data are expressed as mean ± standard deviation (relative to DMSO control) and analyzed using two-way ANOVA, followed by the Dunnett test. *p < 0.05.

This assay evaluated the inhibition of collagenases of *Clostridium hystoliticum* by RuDiOBn and rutin. RuDiOBn significantly inhibited collagenase activity over 24 h, inhibiting 55% (band 1) and 47% (band 2) collagenase, and rutin 36% (band 1) and 58% (band 2), when compared with negative control (DMSO), which was used to observe 100% collagenase activity, and this makes RuDiOBn a possible inhibitor of MMPs involved in aging (Figure 5). Rutin has been cited in the literature as an inhibitor of the expression of MMP-2 and MMP-9, which aided the improvement of inflammation and improved wound healing in hyperglycemic rats.^31^

MMPs participate in events that trigger skin aging, and these enzymes are responsible for degrading ECM components, especially collagen. With aging, these MMPs may be dysregulated, thus promoting the marked degradation of these components. In this sense, the development of inhibitors of MMPs may represent an important therapeutic strategy for preventing aging of the skin.^32^

## 4 Conclusions

The benzylated derivative of rutin (RuDiOBn) showed inhibitory activity against the formation of advanced glycation end products in collagen via the glyoxal pathway, increased the stimulation of fibroblast cell proliferation and migration, stimulated collagen synthesis, and inhibited the proliferation of metalloproteinases. The results of this study point to RuDiOBn as an effective asset for future cosmetic applications aimed at skin aging. The effects could be more pronounced when the compound is incorporated into a formulation in order to enhance cosmetic effects, for example, those related to skin aging, such as glycation of collagen or wrinkles caused by facial expression.

## Supporting Information Available

Information on the high-resolution mass spectra of the RuDiOBn sample with a base peak at *m*/*z* 481, the 1H NMR spectrum (500 MHz, DMSO-d6), where the hydrogen rings were analyzed and the HMBC contour map (1H 500 MHz, 13C 125 MHz, DMSO-d6) of RuDiOBn (PDF)
