# Supplementary material for: Synthesis of a New Benzylated Derivative of Rutin and Study of Its Cosmetic Applications
Source: ACS Omega. 2025 Feb 28;10(9):8883–90. doi: 10.1021/acsomega.4c04908 (PMC11904437; doi:10.1021/acsomega.4c04908)
Supplement: Supplementary file 1 — ao4c04908_si_001.pdf [file ao4c04908_si_001.pdf]

Synthesis of a New Benzylated Derivative of Rutin and Study of Their Cosmetic Applications

Bárbara Janaína Paula da Silva[a], Ana Cristina da Silva Pinto[a], Larissa Barbosa Borges[a], Edinilze Souza Coelho Oliveira[b], Jullio Kennedy Castro Soares[c], Lívia Soman de Medeiros[c], Fernanda Guilhon-Simplicio[a] and Emersom Silva Lima[a,*]

[a]Faculty of Pharmaceutical Sciences, Federal University of Amazonas, 69077-000, Manaus-AM, Brazil.
[b]Nucleus of Amazonian Micromolecules Studies, Institute of Exact Sciences, Federal University of Amazonas, 690770-000, Manaus-AM, Brazil.
[c]Institute of Environmental, Chemical and Pharmaceutical Sciences, Federal University of São Paulo, 09972-270, Diadema-SP, Brazil.

Author Information:

1- Bárbara Janaína Paula da Silva; E-mail: barbaraa.jpaula@gmail.com

2- Ana Cristina da Silva Pinto; E-mail: anacristinadsp@gmail.com

3- Larissa Barbosa Borges; E-mail: larissa.b.borges@gmail.com

4- Edinilze Souza Coelho Oliveira; E-mail: edinilzeo@gmail.com

5- Jullio Kennedy Castro Soares; E-mail: jullio.kennedy@unifesp.br

6- Lívia Soman de Medeiros; E-mail: livia.soman@unifesp.br

7- Fernanda Guilhon Simplicio; E-mail: guilhon_simplicio@ufam.edu.br

8- Emersom Silva Lima; E-mail: eslima@ufam.edu.br

***Corresponding author:** Emersom S. Lima. Faculty of Pharmaceutical Sciences, Federal University of Amazonas. Manaus-AM, Brazil. Av. Rodrigo Otavio, 6200. CEP 69083-020. E-mail: eslima@ufam.edu.br

Tel.: +55 92 988177360

SUPPORTING INFORMATION

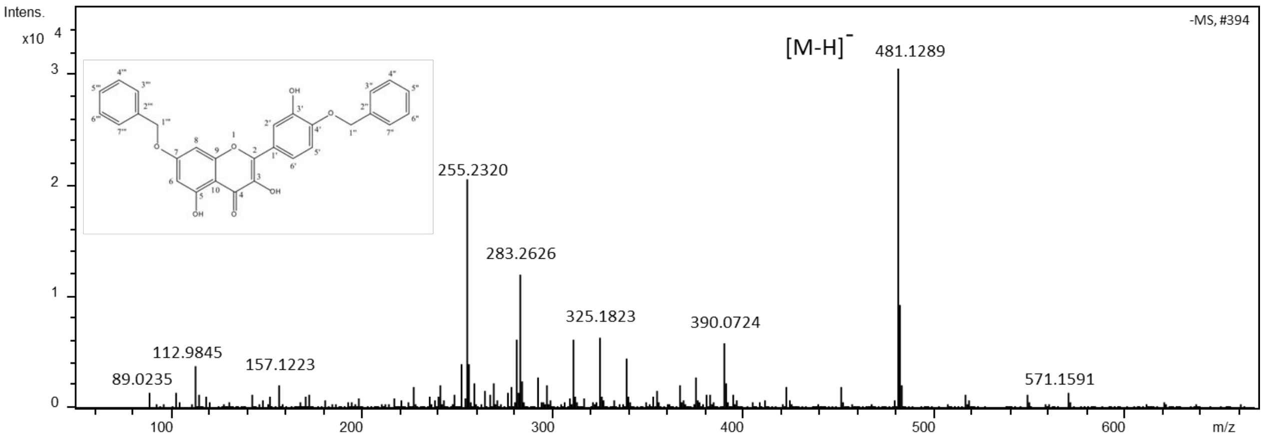

Figure S1. High-resolution Mass spectrum of the RuDiOBn sample with base peak of m/z 481.

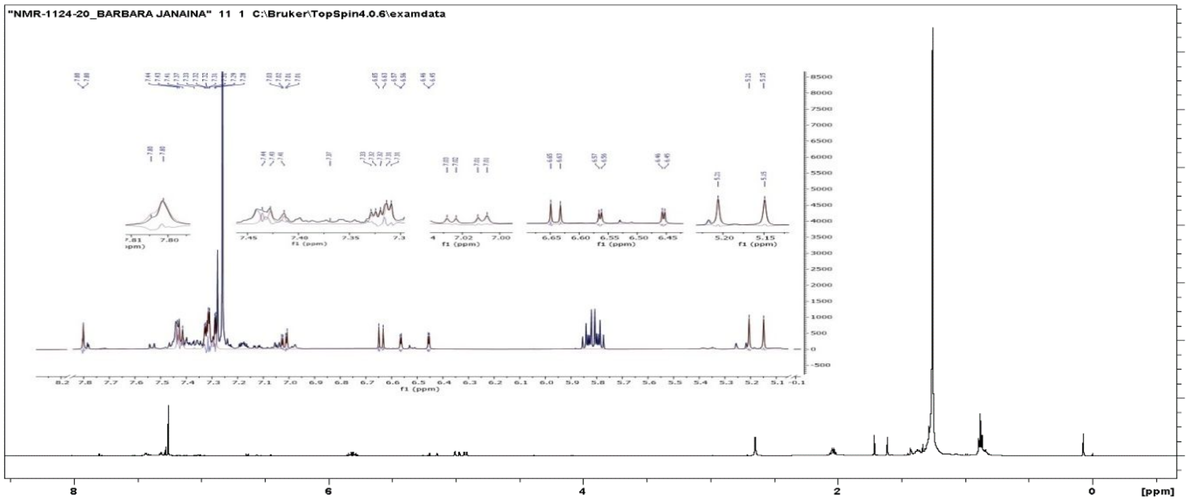

Figure S2. ^{1}H NMR Spectrum (500 MHz, DMSO-d6) of RuDiOBn.

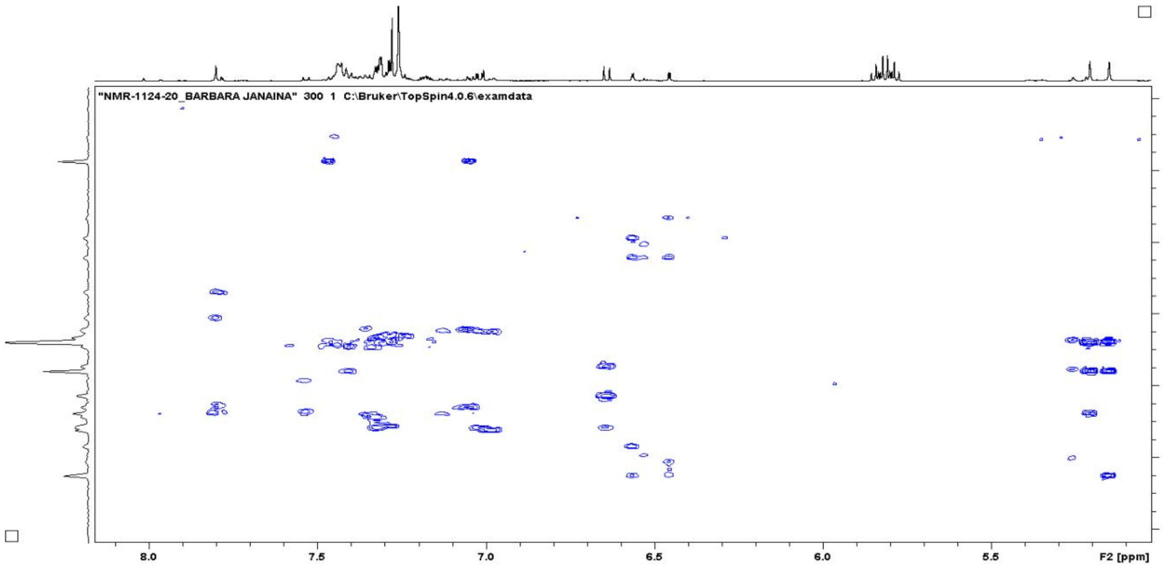

Figure S3. HMBC contour map (^{1}H 500 MHz, ^{13}C 125 MHz, DMSO-d6) of RuDiOBn.
